# Clinical Disease Activity and Power Doppler Changes Observed During a Multimodal Rehabilitation Program in Rheumatoid Arthritis: A Retrospective Longitudinal Observational Study

**DOI:** 10.3390/jcm15208117

**Published:** 2026-10-19

**Authors:** Cristina Octaviana Daia, Madalina Codruta Verenca, Mirela Manea, Elena Valentina Ionescu, Caimac Visarion Danut, Alexandra Maria Poenaru Gilceava, Elena Anca Târtea, Ana Maria Bumbea

**Affiliations:** 1 Faculty of Medicine, Departament 9, Carol Davila University of Medicine and Pharmacy, 050474 Bucharest, Romania; cristina.daia@umfcd.ro; 2 Research Centre in the Medical-Pharmaceutical Field, Medicine and Pharmacy Faculty, “Dunărea de Jos” University of Galati, 800010 Galați, Romania; 3 Department of Physical Medicine and Rehabilitation, Faculty of Medicine, Ovidius University of Constanta, 900470 Constanta, Romania; elena.ionescu@365.univ-ovidius.ro; 4 Faculty of Medicine, Department 1, University of Medicine and Pharmacy of Craiova, 200345 Craiova, Romania; danut.caimac@umfcv.ro (C.V.D.); anamaria.bumbea@umfcv.ro (A.M.B.); 5 Faculty of Medicine, Department 2, University of Medicine and Pharmacy of Craiova, 200249 Craiova, Romania; alexandrapoenaru13@yahoo.com; 6 Faculty of Medicine, Department of Neurology, University of Medicine and Pharmacy of Craiova, 200349 Craiova, Romania; anca.tartea@umfcv.ro

**Keywords:** rheumatoid arthritis, rehabilitation, electrotherapy, physical and rehabilitation medicine, DAS28-ESR, Power Doppler ultrasonography, disease activity

## Abstract

**Background/Objectives:** Rehabilitation in active rheumatoid arthritis (RA) remains challenging, particularly regarding the dosing of physical agent modalities during inflammatory activity. We evaluated changes in clinical disease activity, inflammatory markers, and Power Doppler (PD) activity in patients with RA undergoing individualized multimodal rehabilitation. **Methods:** In this retrospective longitudinal study, 29 patients with RA were assessed at baseline and after 8 weeks. The program comprised 10 supervised sessions of individualized electrotherapy and therapeutic exercise over 2 weeks, followed by 6 weeks of home-based exercise. Background disease-modifying antirheumatic therapy was unchanged; 16 patients received ESR-guided methylprednisolone. Outcomes included DAS28-ESR and its components, C-reactive protein (CRP), and patient-level PD grade across a standardized joint set. **Results:** DAS28-ESR decreased from 6.05 ± 0.87 to 4.03 ± 0.82, with significant reductions in all its components and in CRP (all *p* < 0.001). Patient-level PD grade showed a significant shift toward lower grades, from a median of 2 [IQR 2–3] at baseline to 2 [IQR 1–2] at 8 weeks (*p* < 0.001); 18 patients (62.1%) improved, and none worsened. Changes in DAS28-ESR and PD grade were not significantly correlated (ρ = 0.181, *p* = 0.348). In the 13 patients who did not receive additional glucocorticoids, DAS28-ESR decreased from 5.22 to 3.41 (*p* < 0.001), while median PD grade changed from 2 [IQR 2–3] to 2 [IQR 2–2] (*p* = 0.016). **Conclusions:** Improvements across clinical, laboratory, and ultrasound domains were observed during individualized rehabilitation delivered alongside pharmacological treatment, while the magnitude of clinical and ultrasound changes was not significantly correlated. Because of the absence of a control group, concomitant glucocorticoid and NSAID therapy, and the possibility of regression to the mean, the contribution of rehabilitation cannot be isolated from pharmacological treatment, natural disease course, and other sources of change. Controlled studies of dose-adapted rehabilitation in active RA, prescribed by the rehabilitation physician in collaboration with the rheumatologist, are warranted.

## 1. Introduction

Rheumatoid arthritis (RA) is a chronic systemic inflammatory autoimmune disease characterized by persistent synovitis, progressive structural joint damage, pain, and functional impairment [1].

Despite major advances in pharmacological management with conventional synthetic, biological, and targeted disease-modifying antirheumatic drugs (DMARDs), optimization of physical function and preservation of mobility remain important components of comprehensive disease management [2].

Rehabilitation, particularly therapeutic exercise and physical therapy, represents an important adjunct to pharmacological treatment, with evidence supporting benefits in physical function, disease activity, and quality of life in patients with RA [3].

Assessment of RA activity is inherently multidimensional. Composite clinical indices such as the Disease Activity Score in 28 joints (DAS28) integrate tender and swollen joint counts, the patient’s global assessment, and an inflammatory marker and are widely used to quantify disease activity and therapeutic response [4]. However, clinical composite indices may not fully reflect local synovial inflammatory activity, and discrepancies between clinical findings and persistent subclinical inflammation may occur [5].

Musculoskeletal ultrasonography provides a complementary method for evaluating inflammatory and structural joint involvement in RA [6]. In particular, Power Doppler ultrasonography allows semiquantitative assessment of synovial vascularity and may provide additional information on active synovial inflammation beyond that obtained from clinical examination and systemic inflammatory markers [7]. Consequently, the combined longitudinal assessment of DAS28 and Power Doppler findings may provide a more comprehensive characterization of changes in disease activity [5,8].

Although rehabilitation is increasingly recognized as an important component of comprehensive RA management, evidence regarding its contribution to inflammatory disease activity remains comparatively limited [3,9]. Previous studies of comprehensive rehabilitation have reported favourable clinical and functional outcomes, including changes in disease activity, but the combined longitudinal assessment of clinical, systemic inflammatory, and ultrasound parameters during rehabilitation has received substantially less attention [3,10].

To our knowledge, the longitudinal evolution of composite clinical disease activity, systemic inflammatory markers, and Power Doppler ultrasound findings during structured multimodal rehabilitation in RA has been insufficiently investigated. Therefore, the present study aimed to evaluate changes in clinical disease activity, systemic inflammatory markers, and Power Doppler activity over an 8-week period comprising a supervised multimodal rehabilitation phase followed by home-based therapeutic exercise. A secondary objective was to examine the relationship between changes in DAS28-ESR and Power Doppler activity and to explore whether the observed longitudinal changes differed according to concomitant glucocorticoid exposure.

## 2. Materials and Methods

### 2.1. Study Design and Setting

This was a retrospective, single-centre, longitudinal observational study with a paired pre–post design. Patients were retrospectively identified from existing medical records at the Ambulatory Medical Rehabilitation Department, Craiova, Romania. The clinical, laboratory, and Power Doppler assessments, as well as the ESR-guided pharmacological management, were components of routine clinical practice at the study centre and had been implemented independently of and prior to the conception of the present research. No study-specific prospective assessment or treatment protocol was introduced for the purposes of this investigation.

Eligible records corresponded to patients with rheumatoid arthritis (RA) who had completed the entire 8-week clinical management and follow-up period by 30 June 2026 and for whom complete data required for the present analysis were available. Each patient had been assessed at two time points as part of routine clinical care: at baseline, before initiation of the rehabilitation program (T1), and at the end of the 8-week period (T2). Thus, for all 29 patients included in the study, both T1 and T2 assessments had been completed by 30 June 2026, before initiation of the retrospective research analysis.

The study is reported in accordance with the Strengthening the Reporting of Observational Studies in Epidemiology (STROBE) statement [11]; the completed checklist is provided in Table S1.

### 2.2. Participants

Patients were identified from the clinical practice of the attending physical and rehabilitation medicine (PRM) physician; no external referral pathway was involved. Inclusion criteria were age ≥ 18 years; a diagnosis of RA previously established by a rheumatologist according to the 2010 American College of Rheumatology/European League Against Rheumatism (ACR/EULAR) classification criteria [12]; treatment with a conventional synthetic or biological DMARD for at least 6 months; absence of contraindications to the prescribed rehabilitation procedures; absence of another identifiable cause of the inflammatory syndrome; and completion of the supervised rehabilitation sessions, together with documented, patient-reported performance of the prescribed home-based exercises, as recorded in individual patient notebooks and physiotherapist follow-up records, with complete clinical, laboratory, and ultrasound data at T1 and T2.

Exclusion criteria were decompensated comorbidities contraindicating physical rehabilitation (including decompensated cardiac, renal, respiratory, or hepatic failure), active malignancy, active infection, extensive skin lesions precluding the application of physical modalities, and other concomitant autoimmune diseases. Patients were retrospectively selected from the available medical records according to the eligibility criteria described above and the availability of complete clinical, laboratory, treatment, and ultrasound data at both T1 and T2. The final analytical sample comprised 29 patients with complete records fulfilling these criteria. A comprehensive screening log of all patients with RA evaluated at the department during the study period was not available; therefore, the total number of potentially eligible patients and the exact number and reasons for records not included in the analytical dataset could not be reliably reconstructed retrospectively.

### 2.3. Multimodal Rehabilitation Program

The rehabilitation program comprised 10 supervised sessions administered on weekdays (Monday to Friday) over 2 weeks, followed by a 6-week home-based therapeutic exercise program. During the supervised phase, individualized therapeutic exercise was combined with physical agent modalities selected according to the anatomical site, the predominant clinical manifestations, and the presence or absence of local inflammatory signs. All interventions were prescribed by the PRM physician. Supervised exercise therapy was delivered by two physiotherapists experienced in rheumatological rehabilitation, following the same general therapeutic framework. The rehabilitation program was standardized at the level of its overall schedule and therapeutic principles, while patient-level exercise prescription was individualized according to the anatomical distribution of joint involvement, inflammatory activity, functional limitations, and individual tolerance. As the intervention formed part of routine clinical practice rather than a prospectively designed study protocol, no study-specific training of the physiotherapists was undertaken. The therapeutic exercise program consisted of low-intensity active isotonic exercises performed without resistance and within the functional range of motion required for activities of daily living rather than at the maximum range of joint motion. Targeted isometric exercises were also incorporated, particularly for patients with active inflammation, to maintain muscle activation and function while minimizing joint mobilization and mechanical loading. The exercise program was individually adapted according to joint involvement, pain, swelling, and functional status and included active mobilization without resistance, exercises using a therapeutic stick, and unloaded stationary cycling. Patients were instructed in appropriate exercise techniques and joint-protection principles and were prescribed continuation of the individualized exercises learned and practiced during the supervised phase as a home-based program. As individually applicable, the home-based program included functional-range active mobilization, low-load isotonic exercises without external resistance, targeted isometric exercises, therapeutic-stick exercises, and unloaded stationary cycling. The program was prescribed daily, with one session per day lasting approximately 60 min, throughout weeks 3–8. The therapeutic exercise program followed principles consistent with contemporary EULAR recommendations supporting physical activity in people with rheumatoid arthritis and emphasizing adaptation to individual abilities and clinical condition [13]. Because exercise prescription was individualized within routine clinical practice, the available retrospective records did not provide a uniform quantitative exercise dose across patients. Specifically, the number of sets and repetitions, duration of individual exercises, quantitative exercise load, and predefined progression criteria were not systematically recorded and therefore could not be reliably reconstructed retrospectively. Furthermore, descriptors such as “low intensity” and “functional range of motion” reflected the qualitative terminology used in routine clinical practice rather than predefined, quantitatively measured exercise prescription parameters. Consequently, these descriptors should not be interpreted as standardized intensity or range-of-motion thresholds.

The primary aim of the physical therapy program was to preserve functional joint alignment and counteract characteristic deforming tendencies, particularly ulnar deviation and flexion contractures of the fingers, as well as varus and flexion deformities of the knees in the lower limbs.

Electrotherapy was individualized according to clinical presentation. Inflammatory hand involvement was treated with wrist–hand interferential current therapy (IFC; 100 Hz for 10 min followed by 90–100 Hz for 10 min) combined with transcutaneous electrical nerve stimulation (TENS; 20 min). Knee inflammation with suprapatellar bursal effusion was treated with IFC (100 Hz for 5 min followed by 0–100 Hz for 10 min) combined with diadynamic currents (DDC; diphase fixed, 5 + 5 min, and long period, 5 + 5 min). Therapeutic ultrasound (0.5 W/cm^2^, continuous mode, 5 min) was used for knee or shoulder pain without local inflammatory signs. Bicipital tendinitis was treated with IFC combined with low-level laser therapy (3 J/cm^2^, 10 Hz, four application points), and cervicodorsolumbar pain with descending longitudinal galvanic current (20 min). For all electrical currents, intensity was adjusted to the patient’s perception of vibration. Massage was not part of the protocol. Because individual combinations of physical agent modalities were selected according to each patient’s clinical presentation, and modality-specific exposure counts were not systematically retained in the available retrospective dataset, the number of patients receiving each individual electrotherapy modality could not be reliably reconstructed.

Attendance at supervised sessions was recorded for each patient. During the six-week home-based phase (weeks 3–8), patients maintained individual notebooks in which they recorded the exercises performed at home. Physiotherapists received confirmation messages from patients regarding the performance of the prescribed exercises. In addition, approximately every 14 days, physiotherapists conducted telephone interviews during which they specifically asked patients whether they had performed the prescribed exercises and whether they had experienced any difficulties or adverse reactions. Patients’ responses were documented in the corresponding physiotherapy records, which have been retained.

This procedure represented routine clinical follow-up based on patient–physiotherapist communication rather than standardized quantitative adherence monitoring. For retrospective eligibility assessment, completion of the home-based component was determined from the patients’ notebooks and their self-reported confirmation of exercise performance during follow-up. However, actual exercise adherence and the proportion of prescribed exercises completed were not independently verified or systematically quantified.

### 2.4. Concomitant Pharmacological Treatment

All patients were receiving background DMARD therapy at baseline, consisting of methotrexate, leflunomide, etanercept, adalimumab, or combined leflunomide and methotrexate. Background DMARD therapy remained unchanged throughout the 8-week observation period.

Additional systemic glucocorticoid therapy was prescribed according to a pre-existing ESR-based clinical management protocol routinely used at the study centre and not developed specifically for the present study. The specific ESR thresholds and methylprednisolone dosing regimens reflected this local clinical protocol. The broader use of short-term glucocorticoid therapy in RA is supported by current EULAR recommendations [14]. Patients with a baseline ESR of 50–79 mm/h received oral methylprednisolone 32 mg/day for 5 days, and patients with an ESR ≥ 80 mm/h received intravenous methylprednisolone pulse therapy (1 g/day for 3 consecutive days); patients with an ESR < 50 mm/h did not receive additional glucocorticoids. In all treated patients, glucocorticoid therapy was initiated during the first week of the observation period. Glucocorticoid exposure (route, daily dose, duration, and week of initiation) was extracted from the medical records as actually administered.

As part of routine symptomatic management, patients received a short 10-day course of a non-steroidal anti-inflammatory drug (NSAID), consistent with recommendations supporting oral NSAIDs for symptomatic control of pain and stiffness in RA, at the lowest effective dose for the shortest possible duration [15]: naproxen 550 mg twice daily (*n* = 13), aceclofenac 100 mg twice daily (*n* = 10), or extended-release diclofenac 100 mg once daily (*n* = 6).

### 2.5. Clinical and Laboratory Assessment

Clinical and laboratory assessments were performed at T1 and T2 by the attending physician. Clinical disease activity was assessed using the 28-joint tender joint count (TJC28) [16] and swollen joint count (SJC28) [16]. The patient’s global assessment of health (GH) was recorded on a 0–100 mm visual analogue scale [16]. Patients were asked, “How would you rate your health today?”, with 0 representing the best and 100 the worst perceived health status. Although pain may contribute to the patient’s overall perception of health, the assessment reflected overall health status rather than a specific pain dimension and did not distinguish between pain during activity, at rest, or at night. Laboratory assessment included ESR (mm/h) [17], C-reactive protein (CRP; mg/L) [17], rheumatoid factor (RF; IU/mL) [18], and anti-cyclic citrullinated peptide (anti-CCP) antibodies (U/mL) [19]. RF and anti-CCP were considered positive at values > 14 IU/mL [18] and ≥10 U/mL [19], respectively; results recorded as 0 corresponded to seronegative (below-threshold) results.

Disease activity was calculated using the four-variable Disease Activity Score in 28 joints (DAS28-ESR) [4,16,20]:

DAS28-ESR = 0.56 × √TJC28 + 0.28 × √SJC28 + 0.70 × ln(ESR) + 0.014 × GH

where ESR is expressed in mm/h, GH in mm (0–100), and ln denotes the natural logarithm. Disease activity was categorized as remission (<2.6), low (≥2.6 to ≤3.2), moderate (>3.2 to ≤5.1), or high (>5.1). The individual change between assessments (ΔDAS28-ESR) was calculated for each patient. Clinical response at T2 was classified according to the European Alliance of Associations for Rheumatology (EULAR) response criteria: a good response was defined as a decrease in DAS28-ESR > 1.2 with a T2 value ≤ 3.2; a moderate response as a decrease > 1.2 with a T2 value > 3.2, or a decrease > 0.6 to ≤1.2 with a T2 value ≤ 5.1; and all other cases as no response [20].

Baseline covariates extracted from the medical records included age, sex, disease duration (years from the rheumatologist’s diagnosis to T1), Steinbrocker radiographic stage [21], comorbidities (arterial hypertension, diabetes mellitus, dyslipidaemia, osteoporosis, thyroid disease, and other documented conditions), and smoking status at T1.

### 2.6. Ultrasound Assessment

Musculoskeletal ultrasonography with Power Doppler (PD) was performed at T1 and T2 by the same physician, who had more than 30 years of experience in ultrasonography and held formal certification in musculoskeletal ultrasonography. Examinations were performed using a MyLab 50 ultrasound system (Esaote S.p.A., Genoa, Italy) equipped with a 12–18 MHz linear-array transducer.

The same standardized set of 28 joints was examined in every patient at both time points: bilateral shoulders, elbows, wrists (radiocarpal joints), metacarpophalangeal joints 1–5, proximal interphalangeal joints 1–5 (including the thumb interphalangeal joint), and knees. This 28-joint set was based on the department’s routine clinical ultrasound assessment protocol and did not correspond to a validated composite ultrasound scoring system.

As part of the sonographer’s routine clinical practice, the ankles were also examined; however, ankle findings were supplementary and were not included in the 28-joint PD outcome analysed in the present study. Power Doppler examinations were performed according to the department’s routine clinical acquisition procedures. The exact numerical values of acquisition parameters, including pulse repetition frequency (PRF), gain, and wall filter, were not retained in the available retrospective records. Consequently, consistency of these settings between T1 and T2 could not be independently verified.

In the three patients with previous knee arthroplasty, the prosthetic knee could not be assessed for synovial PD signal; therefore, it did not contribute to the patient-level PD grade.

PD signal was graded semiquantitatively at each examined joint using the EULAR–OMERACT 0–3 scale [22]: grade 0, no PD signal; grade 1, up to three single spots, one confluent spot plus up to two single spots, or up to two confluent spots; grade 2, signal exceeding grade 1 but present in <50% of the synovial area; and grade 3, signal present in >50% of the synovial area. For each patient and time point, the highest PD grade observed among the standardized 28-joint set was retained as the patient-level PD grade; supplementary ankle findings were not included in this patient-level PD outcome. This approach was used because the maximum PD grade was the patient-level ultrasound measure retained in the available routine clinical records and provided an indicator of the greatest degree of Doppler-detected synovial vascularity present at each assessment. However, as a maximum-grade measure, it does not quantify the distribution or cumulative burden of inflammatory activity across the examined joints. The change between assessments (ΔPD) was calculated, and patients were classified as improved, unchanged, or worsened according to whether the patient-level PD grade decreased, remained unchanged, or increased between T1 and T2. Because the ultrasound examinations were performed as part of routine clinical care, individual joint-level PD grades were not retained in the available retrospective analytical records; only the highest PD grade within the standardized 28-joint set was available for each patient and time point. Consequently, a summed or global joint-level PD score could not be retrospectively reconstructed.

Structural involvement was characterized by the presence or absence of ultrasound-detected bone erosions. As erosive status did not change during the observation period, it was recorded as a single dichotomous variable and was not analysed as a longitudinal outcome. The sonographer was aware of the assessment time point and of the clinical data. Ultrasound images were not archived; therefore, blinded re-reading and assessment of inter- or intra-observer reliability were not possible.

### 2.7. Data Sources, Outcomes, and Bias

Data were extracted retrospectively from the medical records using a standardized extraction form with a predefined codebook. Information not documented in the records was coded as not determined and was not imputed. The primary outcomes were the changes in DAS28-ESR and in patient-level PD grade between T1 and T2. Secondary outcomes were the changes in TJC28, SJC28, GH, ESR, CRP, RF and anti-CCP, the distribution of DAS28-ESR activity categories, and the EULAR response at T2.

Potential sources of bias included confounding by concomitant pharmacological treatment, particularly ESR-guided glucocorticoid therapy and NSAID use; regression to the mean, given that patients were treated during a period of high disease activity; and measurement bias, because clinical and ultrasound assessments were performed by the same non-blinded physician. Confounding by glucocorticoid therapy was addressed through a stratified analysis (Section 2.9); the remaining sources of bias are considered in the Discussion.

### 2.8. Study Size

No a priori sample size calculation was performed; the sample comprised the 29 retrospectively selected patients whose records met the eligibility criteria and contained the complete data required for the present analysis. With 29 paired observations, the study had 80% power to detect a standardized paired effect size (Cohen’s d for paired data) of approximately 0.54 at a two-sided α of 0.05.

### 2.9. Statistical Analysis

Continuous variables were summarized as mean ± standard deviation (SD) or median and interquartile range [IQR], according to their distribution, and categorical variables as absolute frequencies and percentages. Normality of continuous variables and of paired differences was assessed using the Shapiro–Wilk test.

Changes between T1 and T2 were analysed using the paired-samples t-test when paired differences were normally distributed and the Wilcoxon signed-rank test otherwise. For normally distributed paired differences, mean changes are reported with 95% confidence intervals (CIs) calculated using the t distribution. For outcomes analysed non-parametrically, median paired changes are reported with percentile-bootstrap 95% CIs. PD grade was treated as an ordinal variable and analysed using the Wilcoxon signed-rank test. Associations between clinical and ultrasound activity were assessed using Spearman’s rank correlation coefficient (ρ), between DAS28-ESR and PD grade at T1 and at T2 and between their longitudinal changes (ΔDAS28-ESR and ΔPD). For Spearman’s ρ, 95% CIs were estimated using percentile bootstrap with 10,000 resamples. Exploratory correlations were examined between ΔPD and changes in TJC28, SJC28, GH, ESR, and CRP.

To address confounding by glucocorticoid therapy, a stratified analysis was performed according to glucocorticoid exposure, comparing patients who received any additional glucocorticoid therapy (oral or intravenous) with those who did not. Baseline characteristics of the two strata were compared using the independent-samples t-test or Mann–Whitney U test for continuous variables and Fisher’s exact test for categorical variables. Within-stratum T1–T2 changes were tested as described above, and between-stratum differences in the magnitude of change were assessed using the Mann–Whitney U test. As an exploratory analysis, changes were also compared across the three exposure categories (none, oral, intravenous) using the Kruskal–Wallis test. Because glucocorticoid allocation was determined by baseline ESR, the strata did not overlap in baseline ESR, which precluded statistical adjustment for this variable; the stratified analysis was therefore considered descriptive.

Exploratory comparisons of ΔDAS28-ESR and ΔPD across background DMARD categories were performed using the Kruskal–Wallis test, and between patients with and without erosions using the Mann–Whitney U test. No correction for multiple comparisons was applied; secondary and subgroup analyses were regarded as exploratory. All tests were two-sided, and *p* < 0.05 was considered statistically significant. Analyses were performed in Python (version 3.13.5) using pandas (version 2.2.3), NumPy (version 2.3.5), and SciPy (version 1.17.0).

### 2.10. Ethical Considerations

The study was conducted in accordance with the Declaration of Helsinki and was approved by the Ethics Committee of the Ambulatory Medical Rehabilitation Department, Craiova (approval no. 187, 18 August 2026). All clinical care and both T1 and T2 assessments included in the present study had been completed by 30 June 2026, before the study received ethical approval. The clinical and ultrasound data were therefore generated as part of routine patient care and not prospectively collected for the purposes of the present research. At their initial clinical evaluation, patients had provided written informed consent permitting the subsequent use of their medical data for future research. Ethical approval for the present retrospective study was obtained before the retrospective research analysis of these previously collected clinical data was undertaken. All patient data were handled in accordance with applicable confidentiality and data protection requirements.

## 3. Results

### 3.1. Participants

**Figure 1:**
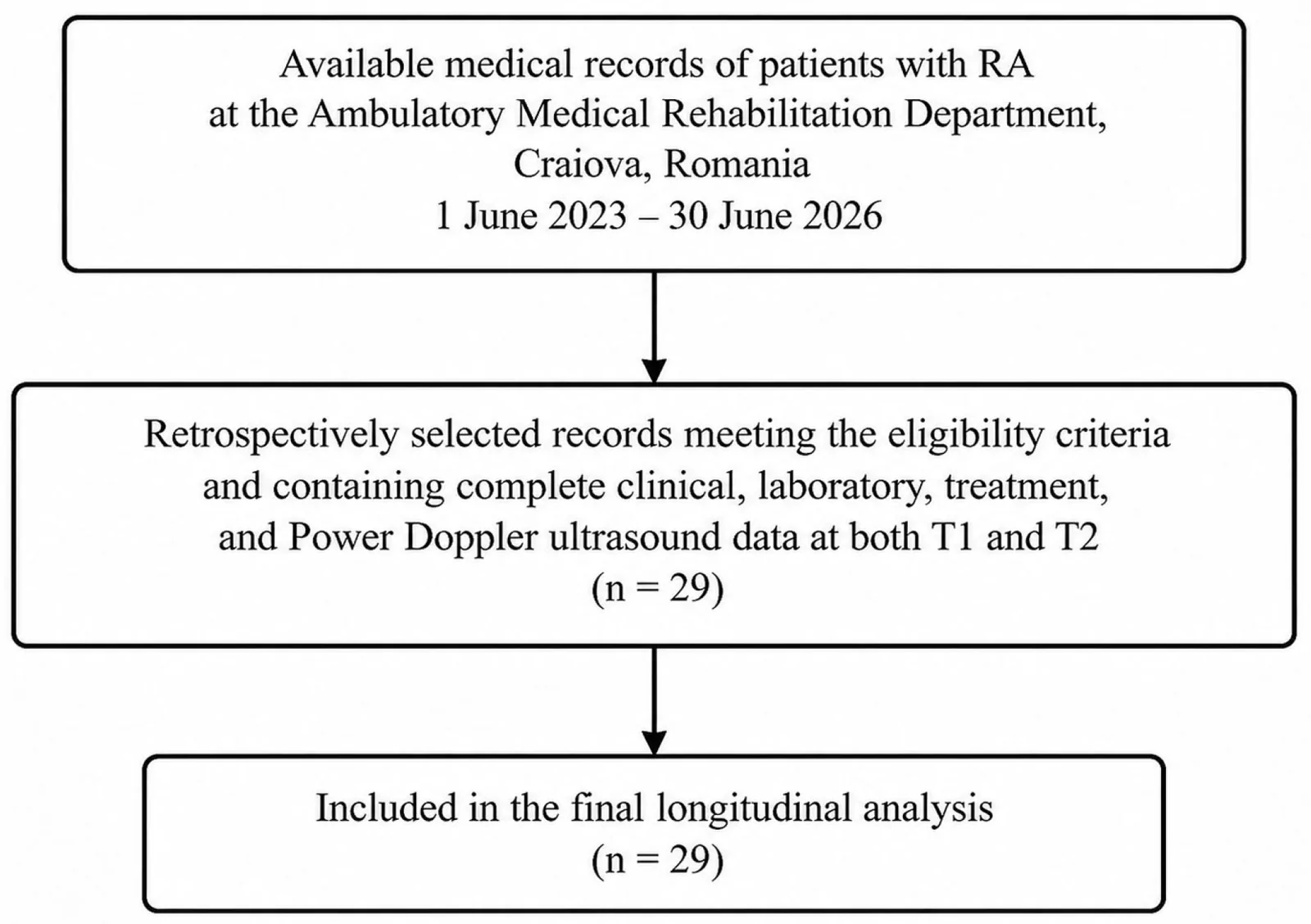
Flow diagram of retrospective record selection and inclusion in the final analysis. The total number of patients with RA evaluated at the department during the study period could not be reliably reconstructed from the available retrospective records.

A total of 29 patients with RA whose medical records fulfilled the eligibility criteria and contained the complete T1 and T2 data required for the study were retrospectively included in the analysis. All 29 patients had completed the 10 supervised rehabilitation sessions and the 8-week clinical follow-up before retrospective selection for the present study. Complete data for the primary and secondary outcomes were available for all included patients. The retrospective selection of records and final analytical sample are presented in Figure 1.

### 3.2. Baseline Characteristics

The study population comprised 23 women (79.3%) and 6 men (20.7%), with a mean age of 47.4 ± 9.5 years (range 28–66). The median disease duration was 6.0 [IQR 3.0–8.0] years (range 1–22). Eleven patients (37.9%) had Steinbrocker stage II, 15 (51.7%) stage III, and 3 (10.3%) stage IV disease, and ultrasound-detected erosions were present in 19 (65.5%).

At least one comorbidity was documented in 15 patients (51.7%), most frequently arterial hypertension (*n* = 12; 41.4%), dyslipidaemia (*n* = 10; 34.5%), osteoporosis, chronic ischaemic heart disease, and knee osteoarthritis (*n* = 9 each; 31.0%). Three patients (10.3%) had undergone knee arthroplasty. Rheumatoid hip involvement (coxitis) was documented in one patient (3.4%). No patient had documented diabetes mellitus or thyroid disease. Six patients (20.7%) were current smokers.

Background therapy consisted of methotrexate in 9 patients (31.0%), leflunomide in 8 (27.6%), etanercept in 6 (20.7%), adalimumab in 5 (17.2%), and combined leflunomide and methotrexate in 1 (3.4%). Additional glucocorticoid therapy was administered to 16 patients (55.2%): oral methylprednisolone to 7 (24.1%) and intravenous methylprednisolone pulse therapy to 9 (31.0%); the remaining 13 patients (44.8%) received no additional glucocorticoids. All patients received a 10-day NSAID course (naproxen, *n* = 13; aceclofenac, *n* = 10; diclofenac, *n* = 6).

Baseline disease activity was predominantly high. The mean DAS28-ESR was 6.05 ± 0.87; 25 patients (86.2%) had high, and 4 (13.8%) moderate disease activity, and none had low disease activity or remission. The median ESR was 50 [IQR 40–80] mm/h. Patient-level PD grade was 3 in 13 patients (44.8%), 2 in 14 (48.3%), and 1 in 2 (6.9%); no patient had grade 0. Baseline characteristics are presented in Table 1.

Because glucocorticoid therapy was allocated according to baseline ESR, the two exposure strata did not overlap in baseline ESR (30–40 mm/h in patients without glucocorticoids vs. 50–120 mm/h in those with glucocorticoids; *p* < 0.001). The strata did not differ significantly in age, sex, disease duration, disease stage, comorbidities, smoking status, or baseline PD grade; however, biological DMARD therapy was more frequent among patients who did not receive glucocorticoids (8/13, 61.5% vs. 3/16, 18.8%; *p* = 0.027) (Table S2).

### 3.3. Changes in Clinical and Laboratory Disease Activity

Clinical disease activity and systemic inflammatory markers improved significantly between T1 and T2 (Table 2).

**Figure 2:**
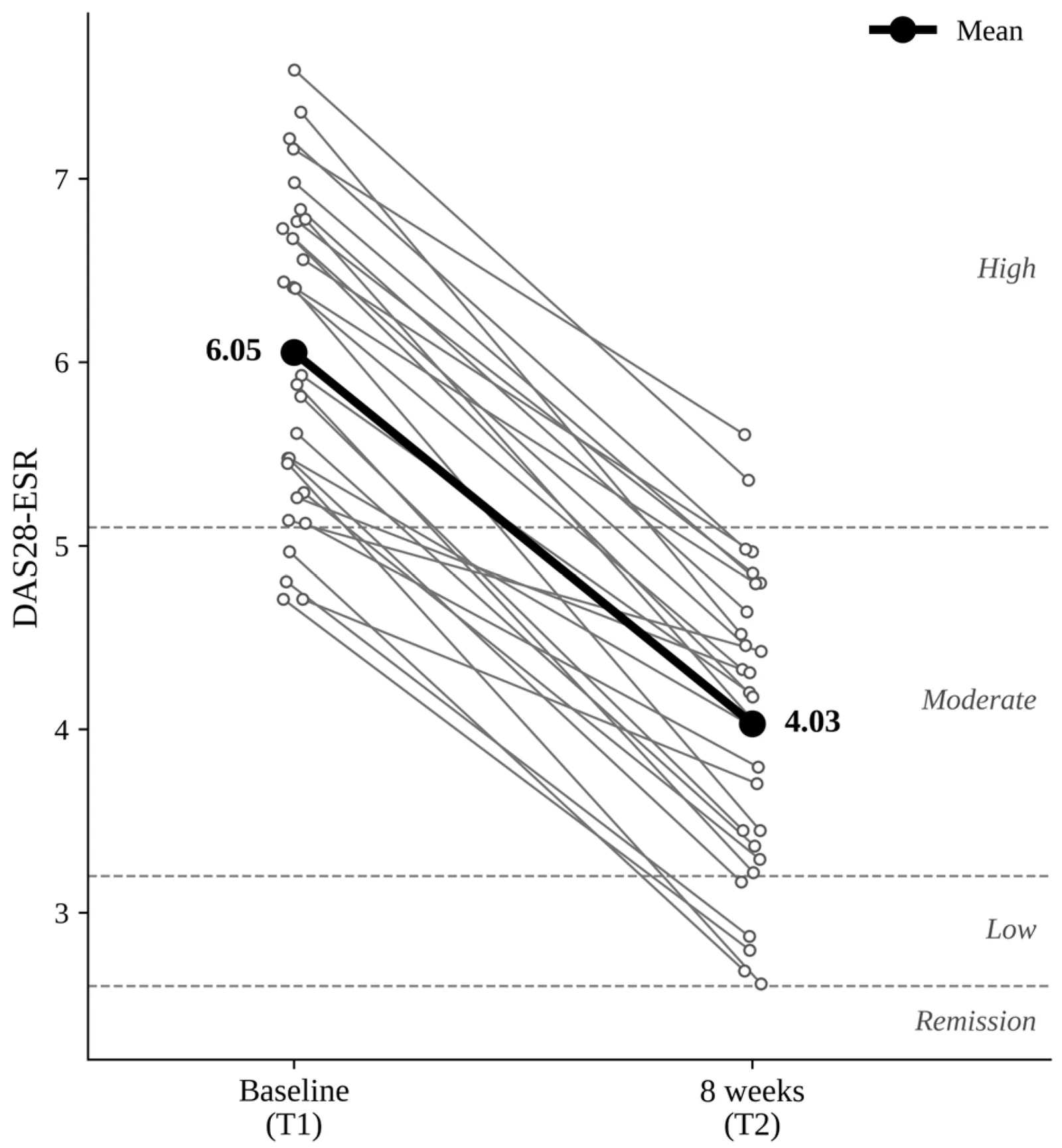
Individual DAS28-ESR trajectories between baseline (T1) and 8 weeks (T2) (*n* = 29). Grey lines and open circles represent individual patients; the bold line indicates the group means. Dashed horizontal lines indicate the DAS28-ESR thresholds of 2.6, 3.2, and 5.1, delimiting remission and low, moderate, and high disease activity.

The median TJC28 decreased from 12 [IQR 8–14] to 4 [IQR 2–6] and the median SJC28 from 6 [IQR 2–8] to 1 [IQR 1–2], while the median GH decreased from 60 [IQR 50–70] mm to 30 [IQR 20–30] mm (all *p* < 0.001). The median ESR decreased from 50 [IQR 40–80] to 30 [IQR 20–30] mm/h and the median CRP from 32 [IQR 16–64] to 16 [IQR 8–24] mg/L (both *p* < 0.001). The mean DAS28-ESR decreased from 6.05 ± 0.87 to 4.03 ± 0.82, corresponding to a mean reduction of 2.02 points (*p* < 0.001). Individual DAS28-ESR trajectories are shown in Figure 2.

### 3.4. Disease Activity Categories and EULAR Response

The distribution of DAS28-ESR activity categories shifted substantially during follow-up (Table 3). At 8 weeks, 2 patients (6.9%) remained in the high disease activity category, whereas 22 (75.9%) had moderate and 5 (17.2%) low disease activity; no patient met the DAS28-ESR remission criterion at either assessment. According to the EULAR response criteria, 5 patients (17.2%) achieved a good response and 24 (82.8%) a moderate response; no patient was classified as a non-responder. The distribution of disease activity categories at T1 and T2 and the EULAR response at T2 are presented in Table 3.

### 3.5. Changes in Power Doppler Activity

Patient-level PD grade, assessed across the standardized 28-joint set at both time points, showed a significant shift toward lower grades, from a median of 2 [IQR 2–3] at T1 to 2 [IQR 1–2] at T2 (Wilcoxon signed-rank test, *p* < 0.001). PD grade decreased in 18 patients (62.1%) and remained unchanged in 11 (37.9%); no patient showed an increase. Among the 13 patients with grade 3 at baseline, 2 improved to grade 1, 9 to grade 2, and 2 remained at grade 3. Among the 14 patients with grade 2, 6 improved to grade 1, and 8 remained unchanged. Of the 2 patients with grade 1, one improved to grade 0, and one remained unchanged. Changes in patient-level PD grade are summarized in Table 4.

**Figure 3:**
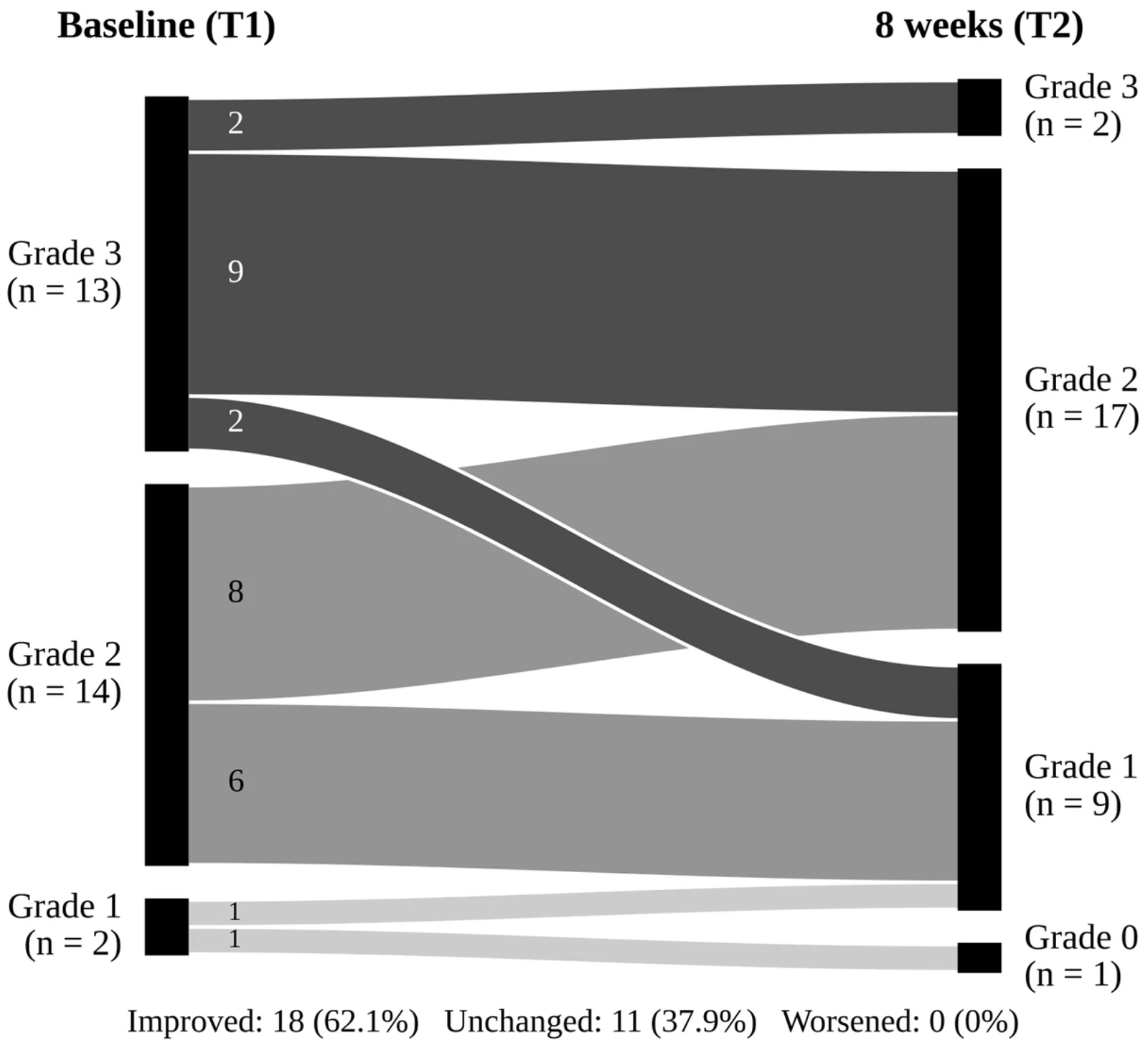
Alluvial plot of individual transitions in patient-level Power Doppler grade between baseline (T1) and 8 weeks (T2) (*n* = 29). Band width is proportional to the number of patients; band shading indicates the baseline grade.

Individual transitions in patient-level PD grade between T1 and T2 are illustrated in Figure 3.

### 3.6. Relationship Between Clinical Disease Activity and Power Doppler Activity

At baseline, DAS28-ESR showed a positive but non-significant correlation with PD grade (ρ = 0.342, *p* = 0.070), and no significant correlation was observed at 8 weeks (ρ = 0.108, *p* = 0.579). Although the percentile bootstrap 95% confidence interval for the baseline correlation marginally excluded zero (0.006–0.625), the two-sided Spearman correlation test did not reach statistical significance. This discrepancy reflects differences between the inferential procedures and warrants cautious interpretation, particularly given the small sample size and tied ordinal PD observations.

ΔDAS28-ESR was not significantly correlated with ΔPD (ρ = 0.181, *p* = 0.348), and no significant correlations were found between ΔPD and changes in TJC28, SJC28, GH, ESR, or CRP (all *p* > 0.05). Thus, although both measures decreased at the group level, the magnitude of individual changes was not significantly correlated. The correlations between DAS28-ESR and patient-level PD grade are summarized in Table 5.

### 3.7. Stratified Analysis According to Glucocorticoid Exposure

Sixteen patients (55.2%) received additional glucocorticoid therapy (oral, *n* = 7; intravenous, *n* = 9), whereas 13 (44.8%) did not. At baseline, mean ESR was 36.2 mm/h in patients without glucocorticoids and 77.8 mm/h in those with glucocorticoids, whereas median PD grade was 2 [IQR 2–3] in patients without glucocorticoids and 2.5 [IQR 2–3] in those receiving glucocorticoids. Baseline characteristics according to additional glucocorticoid exposure are presented in Table S2.

Among the 13 patients who did not receive glucocorticoids, mean DAS28-ESR decreased from 5.22 ± 0.36 to 3.41 ± 0.61 (*p* < 0.001), mean ESR from 36.2 to 19.2 mm/h (*p* < 0.001), mean CRP from 15.8 to 9.2 mg/L (*p* = 0.031), and median PD grade from 2 [IQR 2–3] to 2 [IQR 2–2] (*p* = 0.016). Among the 16 patients who received glucocorticoids, mean DAS28-ESR decreased from 6.73 ± 0.48 to 4.54 ± 0.60 (*p* < 0.001), mean ESR from 77.8 to 33.8 mm/h (*p* < 0.001), mean CRP from 59.8 to 30.5 mg/L (*p* < 0.001), and median PD grade from 2.5 [IQR 2–3] to 2 [IQR 1–2] (*p* = 0.001). TJC28, SJC28, and GH also decreased significantly in both strata (all *p* < 0.001). No statistically significant between-stratum difference was demonstrated for the change in DAS28-ESR (−1.82 vs. −2.19; *p* = 0.181) or PD grade (median paired change: −1 [95% CI −1 to 0] in both strata; *p* = 0.518). Given the small sample size, these findings should not be interpreted as evidence of equivalence between the glucocorticoid strata. Reductions in ESR (−16.9 vs. −44.1 mm/h) and CRP (−6.5 vs. −29.3 mg/L) were larger in the glucocorticoid stratum (both *p* < 0.001), which had higher baseline systemic inflammatory activity. In the no-glucocorticoid stratum, the mean paired CRP change was −6.5 mg/L, whereas the median paired change was 0 mg/L, as reported in Table 6. These estimates differ because they summarize the distribution of individual changes using different measures of central tendency. In the exploratory three-category comparison, no statistically significant differences were demonstrated for ΔDAS28-ESR (none, −1.82; oral, −1.93; intravenous, −2.39; *p* = 0.069) or ΔPD (*p* = 0.701).

Changes in disease activity, systemic inflammatory markers, and patient-level PD grade according to glucocorticoid exposure are summarized in Table 6.

### 3.8. Exploratory Analyses According to Background Therapy and Erosive Status

The magnitude of DAS28-ESR improvement did not differ significantly across background DMARD categories (Kruskal–Wallis test, *p* = 0.186), nor did the change in PD grade (*p* = 0.581). No significant differences were observed between patients with (*n* = 19) and without (*n* = 10) ultrasound-detected erosions in baseline or 8-week DAS28-ESR, baseline or 8-week PD grade, or the magnitude of change in either measure (all *p* > 0.05). Given the small number of patients in individual categories, these analyses are exploratory.

### 3.9. Changes in Serological Markers

At baseline, 26 patients (89.7%) were RF-positive, and 19 (65.5%) were anti-CCP-positive; 3 patients (10.3%) were seronegative for both antibodies, and serological status did not change during follow-up. RF levels showed a small but statistically significant decrease, from a median of 78 [IQR 64–84] IU/mL at T1 to 70 [IQR 60–80] IU/mL at T2 (Wilcoxon signed-rank test, *p* = 0.002), whereas anti-CCP levels remained stable (median 80 [IQR 0–120] U/mL at both time points; *p* = 0.106). Changes in serological markers between T1 and T2 are summarized in Table 7.

## 4. Discussion

This retrospective longitudinal observational study evaluated changes in clinical disease activity, systemic inflammatory markers, and ultrasound PD activity in patients with RA who were retrospectively identified from completed medical records according to the predefined eligibility criteria and who had undergone a multimodal rehabilitation program within routine clinical management. The main findings were a significant improvement in clinical disease activity and inflammatory markers over the 8-week observation period, accompanied by a significant reduction in PD activity, with no patient showing worsening of PD grade. However, the magnitude of improvement in DAS28-ESR was not significantly correlated with the change in PD grade. Significant improvements in DAS28-ESR, its components, CRP, and PD grade were also observed among the 13 patients who did not receive additional glucocorticoid therapy, although the observational design does not allow the effects of rehabilitation to be separated from those of background pharmacological treatment, concomitant NSAID use, regression to the mean, natural disease variability, or other potential confounders.

### 4.1. Changes in Clinical Disease Activity and Rehabilitation Approach

The marked improvement in clinical disease activity observed during follow-up is particularly relevant given the high baseline inflammatory burden of the cohort. DAS28-ESR decreased from 6.05 ± 0.87 to 4.03 ± 0.82, and the favourable evolution was consistent across multiple disease activity domains rather than being confined to the composite score, with parallel improvements in tender and swollen joint counts, general health assessment, and systemic inflammatory markers. The shift from high toward moderate and low disease activity was also observed during follow-up. However, these findings should be interpreted in the context of the retrospective selection of patients who completed the rehabilitation program and had complete follow-up data.

Nevertheless, most patients remained in the moderate disease activity category, and none achieved remission, indicating substantial improvement rather than achievement of the recommended therapeutic target of remission or low disease activity [14].

Pharmacological treatment undoubtedly contributed to this evolution.

More than half of the patients (55.2%) received a short course of methylprednisolone during the first week of the observation period, and all patients received a 10-day NSAID course; both treatments may have contributed to the observed reductions in joint tenderness and swelling, patient-reported global health scores, and acute-phase reactants within the time frame studied. Background DMARD therapy remained unchanged, and significant improvements were also observed in the 13 patients who did not receive additional glucocorticoids. However, this observation does not isolate an effect of rehabilitation, because these patients continued background DMARD therapy, all received NSAIDs, and biological DMARD use was more frequent in the no-glucocorticoid stratum. Evidence nevertheless supports exercise and rehabilitation as complementary components of pharmacological management in RA [9,23], but the independent contribution of rehabilitation to the changes observed in the present cohort cannot be established within this observational design.

### 4.2. Changes in Power Doppler Activity

An important finding was the significant reduction in PD activity over the same observation period. Patient-level PD grade showed a significant shift toward lower grades, from a median of 2 [IQR 2–3] at T1 to 2 [IQR 1–2] at T2, with 18 of 29 patients (62.1%) improving and no patient worsening. Because the same standardized joint set was examined in every patient at both time points, with constant Doppler settings, these changes cannot be explained by differences in the joints assessed.

This finding is relevant because PD provides information on synovial vascularity and therefore represents an imaging dimension of inflammatory activity distinct from the clinical components incorporated into DAS28 [24]. The parallel reduction in clinical disease activity, systemic inflammatory markers, and PD activity suggests that the improvement observed during follow-up extended across clinical, laboratory, and imaging domains.

These ultrasound findings nevertheless require cautious interpretation. The study was not designed to determine whether the reduction in PD activity was attributable specifically to rehabilitation. All patients continued background DMARD therapy and used NSAIDs, while additional glucocorticoids were administered to patients with greater baseline inflammatory activity. Both DMARD and glucocorticoid therapy may influence PD-detected synovial activity [25,26]. Moreover, ultrasound was performed by the same physician who performed the clinical assessment, who was aware of the time point, and images were not archived for blinded re-reading; measurement bias favouring improvement therefore cannot be excluded. The absence of a control group receiving comparable pharmacological management without rehabilitation prevents causal attribution of the PD changes to the rehabilitation program.

### 4.3. Relationship Between DAS28-ESR and Power Doppler Activity

One of the findings was that the association between clinical and ultrasound changes at the individual level did not reach statistical significance. Although both DAS28-ESR and PD activity decreased significantly for the cohort as a whole, baseline DAS28-ESR was only weakly to moderately associated with PD grade and did not reach statistical significance (ρ = 0.342, *p* = 0.070). No significant association was observed at follow-up (ρ = 0.108, *p* = 0.579), and ΔDAS28-ESR was not significantly correlated with ΔPD (ρ = 0.181, *p* = 0.348).

These findings are consistent with previous studies reporting discordance between clinical disease activity and ultrasound-detected synovial inflammation in RA [27,28]. Clinical composite scores and PD ultrasonography may therefore capture partially different but complementary dimensions of disease activity. Changes in PD grade were also not significantly correlated with changes in TJC28, SJC28, GH, ESR, or CRP, further indicating that improvement across clinical, laboratory, and ultrasound domains may not occur proportionally at the individual-patient level. This potential discordance may have practical relevance for disease monitoring and supports the integration of clinical composite indices, inflammatory biomarkers, and musculoskeletal ultrasound, particularly when clinical and imaging findings diverge [29,30].

Nevertheless, the absence of statistically significant correlations in a cohort of 29 patients should not be interpreted as evidence of true independence between these measures. The limited sample size reduces statistical power, and the patient-level PD grade, which condenses information from 28 joints into the highest grade observed, is a four-level ordinal variable with a restricted range that may not capture improvement in joints other than the most active one.

### 4.4. Influence of Glucocorticoid Therapy

The stratified analysis according to glucocorticoid exposure provides additional context for interpreting the longitudinal findings. Glucocorticoids were allocated according to an ESR-based protocol, so that treated patients had markedly higher baseline ESR, with no overlap between strata (30–40 vs. 50–120 mm/h). Because glucocorticoids can reduce both clinical inflammatory activity and PD-detected synovial vascularity [31], and because ESR is itself a component of DAS28-ESR, glucocorticoid exposure represents an important potential confounder, particularly for the change in DAS28-ESR.

Significant reductions in DAS28-ESR, its components, CRP, and PD grade were observed not only in glucocorticoid-treated patients but also in the 13 patients who did not receive additional glucocorticoids, in whom mean DAS28-ESR decreased from 5.22 to 3.41 and median PD grade changed from 2 [IQR 2–3] to 2 [IQR 2–2]. No statistically significant between-stratum difference was demonstrated in the magnitude of change in DAS28-ESR (*p* = 0.181) or PD grade (*p* = 0.518); however, given the small sample size and limited statistical power, these findings should not be interpreted as evidence of equivalence between strata. Similarly, no statistically significant gradient was demonstrated across the three glucocorticoid exposure categories. These findings indicate only that longitudinal improvement was also observed in patients who did not receive additional glucocorticoids; they do not isolate an effect of rehabilitation, because glucocorticoid allocation was non-randomized, background pharmacological treatment was continued, all patients received NSAIDs, and biological DMARD use was more frequent in the no-glucocorticoid stratum. As expected, reductions in ESR and CRP were larger in the glucocorticoid stratum, consistent with both the higher baseline values and the pharmacological effect of glucocorticoids on acute-phase reactants.

Two characteristics of the strata are relevant to this interpretation. First, baseline PD grade was broadly comparable between the two strata, with a median of 2 [IQR 2–3] in patients who did not receive additional glucocorticoids and 2.5 [IQR 2–3] in those who received glucocorticoids. Thus, the baseline difference in PD activity between the strata was less pronounced than the difference in systemic inflammatory activity reflected by ESR. Second, patients who did not receive glucocorticoids had lower baseline ESR and were more frequently receiving biological DMARDs (61.5% vs. 18.8%), which may itself have influenced their trajectory of disease activity. Conversely, the markedly elevated baseline ESR in the glucocorticoid stratum makes larger reductions in ESR and DAS28-ESR more likely through regression to the mean.

These findings do not demonstrate an independent effect of rehabilitation. Glucocorticoid allocation was not randomized but determined by baseline inflammatory activity, the non-overlapping ESR distributions precluded statistical adjustment, and all patients continued background DMARD therapy and used NSAIDs. The contribution of rehabilitation therefore cannot be isolated from pharmacological treatment, regression to the mean, natural fluctuations in disease activity, or other unmeasured factors.

### 4.5. Background Therapy, Erosive Status, and Serological Markers

Exploratory analyses did not identify significant differences in DAS28-ESR or PD improvement according to background antirheumatic treatment. Similarly, the presence of ultrasound-detected erosions was not associated with significant differences in baseline or follow-up DAS28-ESR, PD activity, or their longitudinal changes. These findings should be interpreted cautiously because of the small number of patients within individual treatment categories; the study was not powered to compare DMARD or biological treatment strategies, and these analyses should be regarded as exploratory and hypothesis-generating.

RF showed a small but statistically significant reduction during follow-up, whereas anti-CCP levels remained essentially stable. In contrast to the marked changes in clinical disease activity, acute-phase reactants, and PD activity, serological markers showed limited short-term variation over the 8-week observation period. Previous longitudinal observations have similarly shown that changes in RF and anti-CCP during treatment may differ in magnitude and in their relationship with clinical treatment response [32].

### 4.6. Implications for Rehabilitation in Active Rheumatoid Arthritis

The present findings raise the possibility that appropriately prescribed rehabilitation may have a role within the integrated management of patients with active RA, rather than being considered only after inflammatory activity has been controlled. Although the observational design does not allow the contribution of rehabilitation to be separated from pharmacological treatment, the favourable evolution observed across clinical, laboratory, and ultrasound domains provides a rationale for further investigating this approach.

The role of electrotherapy in RA remains controversial. Current ACR recommendations conditionally recommend against electrotherapy because of the limited and low-certainty evidence supporting improvements in pain and physical function, rather than evidence that appropriately prescribed electrotherapy aggravates inflammatory activity [9]. This distinction is particularly relevant when considering rehabilitation during active disease.

From a rehabilitation perspective, the present approach emphasizes dose selection rather than the indiscriminate application of physical modalities. The biological effects of electrotherapy depend on the selected modality, its parameters and dose, the site of application, and the biological state of the target tissue. Accordingly, analgesic, vasculotrophic, and resorptive protocols were selected according to clinical and paraclinical findings and the intended therapeutic effect, with electrical intensity maintained at the perceptual level of vibration during active inflammatory manifestations. The objective was therefore not merely to select an electrotherapy modality, but to individualize its parameters and dose according to inflammatory status and therapeutic objective. Recent evidence on physiotherapeutic interventions in RA supports the potential value of multimodal approaches, while emphasizing the need for better standardized intervention protocols and higher-quality controlled studies [33].

The same principle of individualized prescription guided therapeutic exercise. Isotonic and isometric exercises were combined to preserve functional joint mobility while providing gentle muscle strengthening to support joint stability and alignment and to limit deforming tendencies, without excessive mechanical loading of painful or swollen joints. Targeted isometric hand exercises were also used in selected patients with active local inflammation, with the aim of maintaining muscle activation while minimizing joint mobilization and mechanical loading. Although the present study was not designed to determine the independent effect of this specific exercise component, its use illustrates the individualized adaptation of therapeutic exercise to inflammatory status. Current recommendations support individualized physical activity and therapeutic exercise as integral components of RA management [34].

The present findings should not be interpreted as evidence that electrotherapy or rehabilitation directly suppresses rheumatoid synovitis. Rather, they raise the hypothesis that individually prescribed and appropriately dosed rehabilitation may be integrated into the management of selected patients with active RA without necessarily requiring complete inflammatory control before its initiation. Testing this hypothesis will require controlled studies capable of distinguishing the contribution of rehabilitation from that of pharmacological treatment. Such an approach requires close interdisciplinary collaboration between the rheumatologist and the physical and rehabilitation medicine physician, integrating disease-modifying pharmacological management with rehabilitation prescribed according to clinical and paraclinical inflammatory status, joint involvement, and functional needs.

### 4.7. Strengths and Limitations

The principal strength of this study is the multidimensional longitudinal assessment combining DAS28-ESR and its individual components with systemic inflammatory markers and PD ultrasonography. The paired design allowed clinical, laboratory, and ultrasound changes to be examined within the same patients; ultrasound was performed on the same standardized joint set in all patients at both time points; and glucocorticoid exposure was extracted from the records as actually administered. A further strength is the pragmatic, individualized rehabilitation approach, in which exercise and electrotherapy parameters were prescribed according to clinical and paraclinical inflammatory status, joint involvement, and functional needs, reflecting routine practice.

Several limitations should be acknowledged. First, the retrospective single-centre design, the small sample drawn from the practice of a single physician, and the absence of an a priori sample size calculation limit statistical power and generalizability. In addition, retrospective selection based on completion of the rehabilitation program and availability of complete T1 and T2 records may have introduced selection bias. Because a comprehensive screening log was unavailable, the total number of potentially eligible patients and the reasons for exclusion could not be reliably reconstructed.

The requirement for documented, patient-reported performance of the home-based exercises may have introduced additional selection bias by favouring patients who maintained communication with the rehabilitation team. Although exercise performance was documented through individual patient notebooks, confirmation messages, and physiotherapist-recorded telephone interviews, adherence was not assessed using a standardized quantitative protocol. In addition, the retrospective records did not contain sufficiently standardized information on exercise dose, including sets, repetitions, duration, load, and progression criteria, while qualitative descriptors such as “low intensity” and “functional range of motion” were not defined by measurable thresholds. Consequently, actual adherence and the delivered exercise dose could not be reliably quantified, limiting the reproducibility of the exercise component of the rehabilitation program.

Second, the absence of a control group receiving comparable pharmacological management without rehabilitation prevents causal attribution of the observed improvements to the rehabilitation program, and regression to the mean cannot be excluded in a cohort treated during high disease activity.

Third, the multimodal and individualized nature of the intervention precludes determination of the contribution of individual components. In addition, validated functional outcome measures were not systematically available in the retrospective clinical records, limiting our ability to determine whether the observed clinical, laboratory, and ultrasound changes were accompanied by measurable improvements in physical function or disability. Future prospective studies should incorporate validated functional measures alongside clinical, laboratory, and ultrasound outcomes to provide a more comprehensive assessment of the clinical relevance of rehabilitation. Fourth, concomitant pharmacological treatment represents an important confounder: ESR-guided glucocorticoids were administered to 55.2% of patients, the non-overlapping ESR distributions between strata precluded statistical adjustment, biological DMARD use was unevenly distributed between strata, and all patients received a 10-day NSAID course. Fifth, clinical and ultrasound assessments were performed by the same physician, who was aware of the assessment time point and clinical data, and ultrasound images were not archived, precluding blinded re-reading and assessment of intra- and inter-observer reliability. Therefore, systematic measurement bias favouring apparent improvement cannot be excluded, and the reproducibility of the ultrasound findings could not be independently assessed. An additional limitation concerns the patient-level PD metric. The use of the highest PD grade observed within the standardized joint set resulted in a restricted four-level ordinal outcome (0–3) and necessarily involved loss of joint-level information. This approach may fail to capture improvement occurring in joints other than the joint retaining the highest PD grade. Because individual joint-level PD grades and archived ultrasound images were unavailable retrospectively, a summed or global PD score and a sensitivity analysis using an alternative PD metric could not be performed. The limited sensitivity of this metric may also have attenuated the observed association between changes in DAS28-ESR and PD grade. Future prospective studies should retain joint-level ultrasound data and incorporate validated summed or global PD scoring approaches. The exact numerical values of Power Doppler acquisition parameters, including pulse repetition frequency (PRF), gain, and wall filter, were not retained in the available retrospective records. Consequently, consistency of these settings between T1 and T2 could not be independently verified, limiting the reproducibility of the ultrasound acquisition protocol. Finally, the 8-week follow-up does not allow assessment of the persistence of the observed changes.

Despite these limitations, the concordant group-level improvement across clinical, systemic inflammatory, and PD outcomes, together with the individual-level discordance between DAS28-ESR and PD changes, provides a rationale for controlled studies investigating individually prescribed multimodal rehabilitation as part of the integrated management of active RA, alongside multidimensional disease monitoring.

## 5. Conclusions

In this retrospective longitudinal study, patients with active RA undergoing individualized multimodal rehabilitation alongside pharmacological treatment showed significant improvements across clinical, systemic inflammatory, and PD outcomes over 8 weeks, with no worsening of PD activity. No significant correlation was observed between changes in DAS28-ESR and PD grade, indicating that the magnitude of clinical and ultrasound changes was not significantly correlated. Significant improvements in DAS28-ESR, CRP, and PD grade were also observed in the 13 patients who did not receive additional glucocorticoids, although this subgroup had lower baseline inflammatory activity.

Given the retrospective observational design, small sample size, absence of a control group, concomitant glucocorticoid and NSAID therapy, and potential measurement bias associated with non-blinded clinical and ultrasound assessment, the independent contribution of rehabilitation cannot be established, and the observed longitudinal improvements should be interpreted cautiously. Nevertheless, the findings provide a rationale for controlled investigation of whether individually prescribed, dose-adapted multimodal rehabilitation—including electrotherapy prescribed according to clinical and paraclinical inflammatory status—may be integrated into the management of active RA. Such an approach requires close collaboration between rheumatology and physical and rehabilitation medicine and the integration of clinical, laboratory, and ultrasound assessment for individualized patient management.

## Figures and Tables

**Table 1:** Baseline demographic, clinical, and treatment characteristics of the study population (*n* = 29).

Characteristic	Value
Age, years, mean ± SD (range)	47.4 ± 9.5 (28–66)
Female sex, *n* (%)	23 (79.3)
Disease duration, years, median [IQR] (range)	6.0 [3.0–8.0] (1–22)
Steinbrocker radiographic stage, *n* (%)	
Stage II	11 (37.9)
Stage III	15 (51.7)
Stage IV	3 (10.3)
Ultrasound-detected erosions, *n* (%)	19 (65.5)
Comorbidities, *n* (%)	
Any comorbidity	15 (51.7)
Arterial hypertension	12 (41.4)
Dyslipidaemia	10 (34.5)
Osteoporosis	9 (31.0)
Chronic ischaemic heart disease	9 (31.0)
Knee osteoarthritis	9 (31.0)
Previous knee arthroplasty	3 (10.3)
Diabetes mellitus	0
Thyroid disease	0
Current smoker, *n* (%)	6 (20.7)
Background DMARD therapy, *n* (%)	
Methotrexate	9 (31.0)
Leflunomide	8 (27.6)
Etanercept	6 (20.7)
Adalimumab	5 (17.2)
Leflunomide + methotrexate	1 (3.4)
Additional glucocorticoid therapy, *n* (%)	
None	13 (44.8)
Oral methylprednisolone, 32 mg/day for 5 days	7 (24.1)
Intravenous methylprednisolone, 1 g/day for 3 days	9 (31.0)
NSAID course (10 days), *n* (%)	29 (100)
Naproxen 550 mg twice daily	13 (44.8)
Aceclofenac 100 mg twice daily	10 (34.5)
Diclofenac (extended-release) 100 mg once daily	6 (20.7)
Baseline disease activity	
DAS28-ESR, mean ± SD	6.05 ± 0.87
High disease activity (DAS28-ESR > 5.1), *n* (%)	25 (86.2)
Moderate disease activity (DAS28-ESR 3.2–5.1), *n* (%)	4 (13.8)
ESR, mm/h, median [IQR]	50 [40–80]
Patient-level PD grade, median [IQR]	2 [2–3]

Abbreviations: DAS28-ESR, Disease Activity Score in 28 joints using erythrocyte sedimentation rate; DMARD, disease-modifying antirheumatic drug; ESR, erythrocyte sedimentation rate; IQR, interquartile range; NSAID, non-steroidal anti-inflammatory drug; PD, Power Doppler; SD, standard deviation.

**Table 2:** Changes in clinical disease activity and inflammatory markers between baseline and 8-week follow-up (*n* = 29).

Outcome	Baseline (T1)	8 Weeks (T2)	Change (T2 − T1), Estimate (95% CI)	*p*-Value
TJC28, median [IQR]	12 [8–14]	4 [2–6]	−7.14 (−8.15 to −6.13)	<0.001
SJC28, median [IQR]	6 [2–8]	1 [1–2]	−4 [−5 to −2]	<0.001
GH, mm, median [IQR]	60 [50–70]	30 [20–30]	−30 [−30 to −25]	<0.001
ESR, mm/h, median [IQR]	50 [40–80]	30 [20–30]	−30 [−35 to −20]	<0.001
CRP, mg/L, median [IQR]	32 [16–64]	16 [8–24]	−20 [−24 to −7]	<0.001
DAS28-ESR, mean ± SD	6.05 ± 0.87	4.03 ± 0.82	−2.02 (−2.24 to −1.80)	<0.001

Note: Changes are expressed as T2 − T1. For normally distributed paired differences, changes are presented as mean differences (95% CI); for outcomes analysed nonparametrically, changes are presented as median paired differences [95% CI], with confidence intervals estimated using percentile bootstrap. For TJC28, baseline and follow-up values are summarized as median [IQR], whereas the paired change is reported as a mean difference (95% CI). Paired comparisons were performed using the paired-samples t-test or Wilcoxon signed-rank test, as appropriate. Abbreviations: CI, confidence interval; CRP, C-reactive protein; DAS28-ESR, Disease Activity Score in 28 joints using erythrocyte sedimentation rate; ESR, erythrocyte sedimentation rate; GH, patient’s global assessment of health; IQR, interquartile range; SD, standard deviation; SJC28, 28-joint swollen joint count; TJC28, 28-joint tender joint count.

**Table 3:** DAS28-ESR disease activity categories at baseline and 8-week follow-up and EULAR response at 8-week follow-up (*n* = 29).

Category	Baseline, *n* (%)	8 Weeks, *n* (%)
DAS28-ESR category		
High disease activity	25 (86.2)	2 (6.9)
Moderate disease activity	4 (13.8)	22 (75.9)
Low disease activity	0	5 (17.2)
Remission	0	0
EULAR response		
Good response	—	5 (17.2)
Moderate response	—	24 (82.8)
No response	—	0

Abbreviations: DAS28-ESR, Disease Activity Score in 28 joints using erythrocyte sedimentation rate; EULAR, European Alliance of Associations for Rheumatology.

**Table 4:** Changes in patient-level Power Doppler grade between baseline and 8-week follow-up (*n* = 29).

Transition (T1 → T2)	*n* (%)
Grade 1 → Grade 0	1 (3.4)
Grade 1 → Grade 1	1 (3.4)
Grade 2 → Grade 1	6 (20.7)
Grade 2 → Grade 2	8 (27.6)
Grade 3 → Grade 1	2 (6.9)
Grade 3 → Grade 2	9 (31.0)
Grade 3 → Grade 3	2 (6.9)
Improved	18 (62.1)
Unchanged	11 (37.9)
Worsened	0

Note: Patient-level PD grade was defined as the highest grade observed among the standardized 28-joint set; supplementary ankle findings were not included. Abbreviations: PD, Power Doppler.

**Table 5:** Associations between DAS28-ESR and Power Doppler grade (*n* = 29).

Association	Spearman ρ	95% CI	*p*-Value
DAS28-ESR T1 vs. PD grade T1	0.342	0.006 to 0.625	0.070
DAS28-ESR T2 vs. PD grade T2	0.108	−0.282 to 0.461	0.579
ΔDAS28-ESR vs. ΔPD grade	0.181	−0.207 to 0.542	0.348

Abbreviations: CI, confidence interval; DAS28-ESR, Disease Activity Score in 28 joints using erythrocyte sedimentation rate; PD, Power Doppler; T1, baseline assessment; T2, 8-week assessment; Δ, change from T1 to T2; ρ, Spearman’s rank correlation coefficient. The 95% CIs for Spearman’s ρ were estimated using percentile bootstrap with 10,000 resamples.

**Table 6:** Changes in disease activity, inflammatory markers, and Power Doppler grade between baseline and 8-week follow-up according to glucocorticoid exposure.

Outcome	No GC T1 (*n* = 13)	No GC T2	Δ No GC (95% CI)	Within *p*	GC T1 (*n* = 16)	GC T2	Δ GC (95% CI)	Within *p*	Between Δ *p*
DAS28-ESR	5.22 ± 0.36	3.41 ± 0.61	−1.82 (−2.22 to −1.41)	<0.001	6.73 ± 0.48	4.54 ± 0.60	−2.19 (−2.42 to −1.96)	<0.001	0.181
ESR, mm/h	36.2 ± 5.1	19.2 ± 10.2	−16.9 (−22.5 to −11.3)	<0.001	77.8 ± 24.4	33.8 ± 10.3	−44.1 (−54.4 to −33.7)	<0.001	<0.001
CRP, mg/L	15.8 ± 10.1	9.2 ± 4.7	0 [−8 to 0]	0.031	59.8 ± 32.1	30.5 ± 21.5	−29.3 (−38.1 to −20.4)	<0.001	<0.001
PD grade, median [IQR]	2 [2–3]	2 [2–2]	−1 [−1 to 0]	0.016	2.5 [2–3]	2 [1–2]	−1 [−1 to 0]	0.001	0.518

Note: Continuous variables at T1 and T2 are presented as mean ± SD, except for PD grade, which is presented as median [IQR]. Changes (Δ) are expressed as T2 − T1. For normally distributed paired differences, Δ is presented as mean difference (95% CI), with 95% CIs calculated using the t distribution. For outcomes analysed nonparametrically, Δ is presented as median paired difference [95% CI], with 95% CIs estimated using percentile bootstrap. Within-stratum *p*-values refer to paired-samples t-tests or Wilcoxon signed-rank tests, as appropriate. Between-stratum *p*-values compare the magnitude of change between the two glucocorticoid strata using the Mann–Whitney U test. Abbreviations: CI, confidence interval; GC, glucocorticoids; DAS28-ESR, Disease Activity Score in 28 joints using erythrocyte sedimentation rate; ESR, erythrocyte sedimentation rate; CRP, C-reactive protein; PD, Power Doppler; IQR, interquartile range; SD, standard deviation.

**Table 7:** Changes in serological markers between baseline and 8-week follow-up.

Serological Marker	T1, Median [IQR]	T2, Median [IQR]	*p*-Value
RF	78 [64–84]	70 [60–80]	0.002
anti-CCP	80 [0–120]	80 [0–120]	0.106

Note: Paired comparisons were performed using the Wilcoxon signed-rank test. Positivity thresholds were RF > 14 IU/mL [18] and anti-CCP ≥ 10 U/mL [19]. Abbreviations: anti-CCP, anti-cyclic citrullinated peptide antibodies; IQR, interquartile range; RF, rheumatoid factor; T1, baseline assessment; T2, 8-week assessment.

## Data Availability

The data supporting the findings of this study are available from the corresponding author upon reasonable request. The data are not publicly available due to privacy and confidentiality considerations related to the study participants.

## Supplementary Materials

The following supporting information can be downloaded at: https://www.mdpi.com/article/10.3390/jcm15208117/s1, Table S1. STROBE checklist; Table S2. Baseline characteristics according to additional glucocorticoid therapy.

## Abbreviations

The following abbreviations are used in this manuscript:

ACR	American College of Rheumatology
anti-CCP	Anti-cyclic citrullinated peptide antibodies
CRP	C-reactive protein
DAS28-ESR	Disease Activity Score in 28 joints using erythrocyte sedimentation rate
DDC	Diadynamic currents
DMARD	Disease-modifying antirheumatic drug
ESR	Erythrocyte sedimentation rate
EULAR	European Alliance of Associations for Rheumatology
GC	Glucocorticoid
GH	Patient’s global assessment of health
IFC	Interferential current therapy
IQR	Interquartile range
NSAID	Non-steroidal anti-inflammatory drug
OMERACT	Outcome Measures in Rheumatology
PD	Power Doppler
PRM	Physical and rehabilitation medicine
RA	Rheumatoid arthritis
RF	Rheumatoid factor
SD	Standard deviation
SJC28	28-joint swollen joint count
STROBE	Strengthening the Reporting of Observational Studies in Epidemiology
TENS	Transcutaneous electrical nerve stimulation
TJC28	28-joint tender joint count
