# Supplementary material for: Clinical Disease Activity and Power Doppler Changes Observed During a Multimodal Rehabilitation Program in Rheumatoid Arthritis: A Retrospective Longitudinal Observational Study
Source: J Clin Med. 2026 Oct 19;15(20):8117. doi: 10.3390/jcm15208117 (PMC13705960; doi:10.3390/jcm15208117)
Supplement: Supplementary file 1 [file jcm-15-08117-s001.zip › jcm-4591909-supplementary.pdf]

Table S1. STROBE checklist

STROBE Statement — checklist of items that should be included in reports of cohort studies, completed for: Daia et al., "Clinical Disease Activity and Power Doppler Changes Following a Multimodal Rehabilitation Program in Rheumatoid Arthritis: A Retrospective Longitudinal Observational Study". Section numbers refer to the submitted manuscript; page numbers should be inserted after typesetting.

Item	Section / Topic	STROBE recommendation	Where addressed in the manuscript
1	Title and abstract	(a) Indicate the study's design with a commonly used term in the title or the abstract. (b) Provide in the abstract an informative and balanced summary of what was done and what was found.	**Title ("A Retrospective Longitudinal Observational Study"); Abstract**
2	Background/rationale	Explain the scientific background and rationale for the investigation being reported.	**Section 1, paragraphs 1–5**
3	Objectives	State specific objectives, including any prespecified hypotheses.	**Section 1, final paragraph**
4	Study design	Present key elements of study design early in the paper.	**Section 2.1, first paragraph**
5	Setting	Describe the setting, locations, and relevant dates, including periods of recruitment, exposure, follow-up, and data collection.	**Section 2.1 (Ambulatory Medical Rehabilitation Department, Craiova, Romania; study setting and relevant follow-up dates)**
6	Participants	(a) Give the eligibility criteria, and the sources and methods of selection of participants; describe methods of follow-up.	**Sections 2.1–2.2; Figure 1**
7	Variables	Clearly define all outcomes, exposures, predictors, potential confounders, and effect modifiers.	**Sections 2.3–2.7**
8	Data sources / measurement	For each variable of interest, give sources of data and details of methods of assessment. Describe comparability of assessment methods if there is more than one group.	**Sections 2.3–2.7, including rehabilitation and pharmacological treatment, clinical and laboratory assessments, ultrasound assessment, and retrospective data extraction**
9	Bias	Describe any efforts to address potential sources of bias.	**Section 2.7; Section 4.7**

**10**	Study size	Explain how the study size was arrived at.	**Section 2.8 (available retrospective sample and minimum detectable paired effect size)**
**11**	Quantitative variables	Explain how quantitative variables were handled in the analyses; if applicable, describe which groupings were chosen and why.	**Sections 2.5, 2.6, and 2.9 (DAS28-ESR categories; PD grade treated as ordinal; statistical handling of continuous variables)**
**12**	Statistical methods	(a)–(e) Describe all statistical methods, subgroup analyses, handling of missing data, loss to follow-up, and sensitivity analyses.	**Sections 2.7 and 2.9**
**13**	Participants	(a) Report numbers of individuals at each stage of the study. (b) Give reasons for non-participation. (c) Consider use of a flow diagram.	**Section 3.1; Figure 1**
**14**	Descriptive data	(a) Give characteristics of study participants and information on exposures and potential confounders. (b) Indicate number of participants with missing data for each variable of interest. (c) Summarise follow-up time.	**Sections 3.1–3.2; Table 1; Table S2**
**15**	Outcome data	Report numbers of outcome events or summary measures over time.	**Sections 3.3–3.9; Tables 2–7; Figures 2 and 3**
**16**	Main results	(a) Give unadjusted estimates and, if applicable, confounder-adjusted estimates and their precision. (b) Report category boundaries when continuous variables were categorized.	**Sections 3.3–3.7; Tables 2, 5, and 6 (principal estimates and 95% CIs); Section 2.5 for DAS28-ESR category boundaries**
**17**	Other analyses	Report other analyses done — e.g. analyses of subgroups and interactions, and sensitivity analyses.	**Sections 3.6–3.9; Tables 5–7; Table S2**
**18**	Key results	Summarise key results with reference to study objectives.	**Section 4, first paragraph**
**19**	Limitations	Discuss limitations of the study, taking into account sources of potential bias or imprecision.	**Section 4.7**
**20**	Interpretation	Give a cautious overall interpretation of results considering objectives, limitations, multiplicity of analyses,	**Sections 4.1–4.7; Section 5**

		results from similar studies, and other relevant evidence.	
**21**	Generalisability	Discuss the generalisability (external validity) of the study results.	**Section 4.7**
**22**	Funding	Give the source of funding and the role of the funders.	**Funding statement**

Items 13–17 are reported under Results; items 18–21 under Discussion. An explanation and elaboration article discusses each checklist item and is available at https://www.strobe-statement.org.

Table S2. Baseline characteristics according to additional glucocorticoid therapy.

Characteristic	No GC (n = 13)	GC (n = 16)	p-value
Age, years, mean ± SD	50.5 ± 8.0	44.9 ± 10.2	0.117 [a]
Female sex, n (%)	9 (69.2)	14 (87.5)	0.364 [c]
Disease duration, years, median [IQR]	7.0 [4.5–12.0]	4.8 [2.4–7.3]	0.099 [b]
Disease stage, n (%)			0.864 [b]
Stage II	5 (38.5)	6 (37.5)	
Stage III	7 (53.8)	8 (50.0)	
Stage IV	1 (7.7)	2 (12.5)	
Any comorbidity, n (%)	8 (61.5)	7 (43.8)	0.462 [c]
Arterial hypertension	7 (53.8)	5 (31.3)	0.274 [c]
Dyslipidaemia	5 (38.5)	5 (31.3)	0.714 [c]
Osteoporosis	4 (30.8)	5 (31.3)	1.000 [c]
Chronic ischaemic heart disease	4 (30.8)	5 (31.3)	1.000 [c]
Knee osteoarthritis	5 (38.5)	4 (25.0)	0.688 [c]
Current smoker, n (%)	3 (23.1)	3 (18.8)	1.000 [c]
Biological DMARD, n (%)	8 (61.5)	3 (18.8)	0.027 [c]

**ESR, mm/h, median [IQR] (range)**	**40 [30–40] (30–40)**	**80 [60–82.5] (50–120)**	**< 0.001 [b]**
**Patient-level PD grade, n (%)**			**0.864 [b]**
**Grade 1**	**0**	**2 (12.5)**	
**Grade 2**	**8 (61.5)**	**6 (37.5)**	
**Grade 3**	**5 (38.5)**	**8 (50.0)**	
**DAS28-ESR, mean ± SD**	**5.22 ± 0.36**	**6.73 ± 0.48**	**< 0.001 [a]**

[a] Independent-samples t-test; [b] Mann–Whitney U test; [c] Fisher's exact test. Abbreviations: GC, additional glucocorticoid therapy (oral or intravenous methylprednisolone); DAS28-ESR, Disease Activity Score in 28 joints using erythrocyte sedimentation rate; DMARD, disease-modifying antirheumatic drug; ESR, erythrocyte sedimentation rate; IQR, interquartile range; PD, Power Doppler; SD, standard deviation.
